# Pattern of Mortality among Patients Admitted in Medical Wards in a Tertiary Care Centre of Nepal: A Descriptive Cross-sectional Study

**DOI:** 10.31729/jnma.8784

**Published:** 2024-10-31

**Authors:** Min Chandra Adhikari, Surendra Lal Shrestha, Sarbesh Sharma, Suresh Prasad Nepal

**Affiliations:** 1 Department of Medicine, National Academy of Medical Sciences, Kathmandu, Nepal; 2 Department of General Practice and Emergency Medicine, National Academy of Medical Sciences, Kathmandu, Nepal

**Keywords:** *admission*, *mortality*, *Nepal*, *pattern*

## Abstract

**Introduction:**

Most developing regions of the world are undergoing gradual epidemiological transition resulting in high burden of both communicable and noncommunicable diseases. Morbidity and mortality pattern is a reflection of disease burden. Detailed assessment of these parameters tends to aid in formulating pivotal aspects of in-hospital policies, standards of care and so consequently promoting efficient work systems. The aim of this study is to identify the pattern of mortalities in medical wards in a tertiary care hospital.

**Methods:**

This retrospective descriptive cross-sectional study was conducted in National Academy of Medical Sciences, Bir hospital focusing on all patients admitted to the medical wards (General Medical, Hepatology, Nephrology, Neurology, Endocrinology, Pulmonology, Cardiology and Gastrology) between January 1, 2023 to December 31, 2023 after obtaining ethical approval from Institutional Review Committee (Reference number: 390/2080/81). Total population sampling was done. Data were analyzed using Microsoft Excel and SPSS.

**Results:**

Among 10124 admitted patients in medical wards, mortality was reported in 732 (7.22%). The number of deaths seen in general medical ward was 389 (53.14%) and in hepatology ward was 85 (11.61%). Systemic cause of death due to respiratory diseases was 157 (21.43%) and liver disease was 143 (19.54%).

**Conclusions:**

The respiratory disease is responsible for a higher proportion of deaths among admitted patients.

## INTRODUCTION

The global burden of disease has shifted dramatically over the past 30 years.^1^ More than 60 million people die globally each year showing a shift in the disease paradigm from communicable to non-communicable diseases (NCDs).^2,3^ In settings, such as Nepal, the epidemiological transition from infectious to non-communicable drivers of morbidity and mortality is ongoing, resulting in a double burden of a concurrent high burden of chronic and infectious diseases.^4^ The non-communicable diseases in Nepal were responsible for nearly two-thirds of total deaths in 2015, compared to 29.91% in 1990.^5^

Hospital deaths, while often unavoidable, are seen as an indirect measure of the quality of healthcare services provided by the institution.^6^ So, understanding mortality patterns provides valuable insights into the effectiveness of current healthcare interventions and identifies areas needing improvement. In Nepal, where population-based mortality data are limited, hospital-based mortality analysis becomes crucial.^7^

This study aimed to identify the in-hospital mortality rate of patients admitted in medical wards.

## METHODS

This is a descriptive cross-sectional study conducted at the National Academy of Health Sciences, Bir Hospital, a tertiary care hospital located in Kathmandu, Nepal. A retrospective analysis of the medical record of all mortality in medical wards between January 1 to December 31, 2023 was carried out. Ethical approval was obtained from the institutional review board (Reference Number-390/2080/81). Total population sampling was done. Mortality in the medical wards, which include General Medical, Hepatology, Nephrology, Neurology, Endocrinology, Pulmonology, Cardiology, and Gastroenterology wards, was included in the study. Incomplete records and mortality in the emergency department were excluded from the study. The information was retrieved from the record section, and a standardized proforma was filled up as a part of data collection. The patient's gender, age, the ward where the patient was admitted, and the cause of death were retrieved from the record. The information was tabulated in an Excel sheet. Descriptive analysis was done using SPSS (Statistical Package for Social Sciences), and results were represented in frequency and proportion.

## RESULTS

Among the 10,124 admitted patients, 5558 (54.90%) were male, and 4,566 (45.10%) were female, maintaining a ratio of 1.2 males to every female. Among the admitted patients, 4738 (46.80%) admitted in General Medicine, 1294 (12.78%) in Nephrology, 1412 (13.95%) admitted in Gastrology, 691 (6.83%) in Neurology, 633 (6.25%) in Hepatology ward, 624 (6.16%) in Cardiology, 559 (5.52%) in pulmonology and 173 (1.71%) in endocrinology wards. Among the total admitted patients 87% were recovered and discharged. The number of people leaving against medical advice was very high that is 4.2%.

There were 732 (7.23%) deaths in medical wards, and the proportion of deaths in male was 423 (7.61%) and that in female was 309 (6.76%) (Table 1).

The median age of death was 58 years (IQR: 44-71 years) The median age of mortality of male is 57 years (IQR: 44-70 years). The median age of mortality of female was 60 (IQR: 45-73 years). There were 139 (18.99%) deaths in the age group of 70-80 years and 8 (1.09%) in the age group less than 20 years (Table 2).

**Table 1:** Mortality distribution by wards and gender (n=732).

Wards	Male n (%)	Female n (%)	Total n (%)
General Medicine	223 (30.46)	166 (22.68)	389 (53.14)
Hepatology	55 (7.51)	30 (4.10)	85 (11.61)
Nephrology	28 (3.83)	32 (4.37)	60 (8.20)
Pulmonology	29 (3.96)	25 (3.42)	54 (7.38)
Gastrology	35 (4.78)	19 (2.60)	54 (7.38)
Neurology	28 (3.83)	18 (2.46)	46 (6.28)
Cardiology	22 (3.01)	16 (2.19)	38 (5.19)
Endocrinology	3 (0.41)	3 (0.41)	6 (0.82)

**Table 2:** Mortality distribution by age group and gender (n=732).

Age group (in years)	Male n (%)	Female n (%)	Total n (%)
Less than 20	1 (0.14)	7 (0.96)	8 (1.09)
20 to 30	25 (3.42)	23 (3.14)	48 (6.56)
30 to 40	46 (6.28)	25 (3.42)	71 (9.70)
40 to 50	76 (10.38)	41 (5.60)	117 (15.98)
50 to 60	81 (11.07)	51 (6.97)	132 (18.03)
60 to 70	82 (11.20)	53 (7.24)	135 (18.44)
70 to 80	71 (9.70)	68 (9.29)	139 (18.99)
80 to 90	32 (4.37)	34 (4.64)	66 (9.02)
More than 90	9 (1.23)	7 (0.96)	16 (2.19)

The median hospital stay before death was 5 (IQR:2-9) days. Among total admitted patients 8967 (88.57%) were discharged and 425 (4.19%) left against medical advice (LAMA).

Death due to respiratory system was 157 (21.45%) and that due to poisoning was 7 (0.96%) (Table 3).

**Table 3:** Mortality distribution by age group and gender (n=732).

ICD11 code	System	Number of death n (%)
12	Diseases of respiratory system	157 (21.45)
13	Disease of digestive system: liver diseases	143 (19.54)
01	Certain infection and parasitic dis-eases	91 (12.43)
08	Diseases of nervous system	80 (10.93)
16	Diseases of the genitourinary Sys-tem	77 (10.52)
13	Diseases of digestive system (with-out liver diseases)	76 (10.38)
11	Diseases of circulatory system	68 (9.29)
03	Diseases of blood and blood form-ing organs	14 (1.91)
05	Endocrine, nutritional and metabol-ic diseases	10 (1.37)
02	Neoplasm	9 (1.23)
22	Poisoning	7 (0.96)

## DISCUSSION

There were 732 (7.23%) deaths among patients in the medical ward in our study. Males constituted 57.78% and females 42.22% of the deaths. In contrast to our study, lower mortality rates were observed in developed countries where there is sufficient availability of advanced health services and facilities compared to our country. A retrospective study (2015-2016) conducted in a Canadian tertiary hospital 5% and study conducted in Spain 6 years (Jan 1997-Dec 2002) yrs Hospital de Vic (Barcelona) 5.1%.^8,9^ A study done in Pakistan reported the mortality rate of 6.2% whereas 12.5% mortality rate was reported at Jimma University Specialized Hospital of Ethiopia.^10,11^ Notably, hospital admissions related to liver diseases exhibited the highest mortality rate among all chronic diseases, accounting for 13.4%. This rate surpassed the findings of a previous study conducted in Germany which reported a mortality rate of 9.49% in 2018.^12^ This difference in mortality rate due to the same condition may be due to early diagnosis and treatment and availability of health services. These findings underscore the importance of context when comparing mortality rates and hospital outcomes. Differences in healthcare infrastructure, resource availability, and access to advanced medical services play a crucial role in determining patient outcomes.

Notably, there were a higher number of male admissions and deaths compared to females. Deaths in male was 423 (7.61%) and that in female was 309 (6.76%) medical ward. This finding is similar to a study done in Nigeria where mortality rate was significantly higher in males, (14.3%) than in females (8.4%). This gender difference may be attributed to men being less health-conscious, seeking medical attention only when complications arise.^13^ A study published in the BMC Public Health journal revealed that men tend to utilize health services less frequently than women, often delaying seeking help even when facing serious health problems. The research identified various factors influencing healthcare utilization among men. These factors encompass socio-economic conditions, health-related aspects, and lifestyle choices, such as self-rated health, smoking, drinking, maintaining a healthy weight, use of pain medication, and more.^14^

Among the total admitted patients 88.57% were recovered and discharged. The number of people leaving against medical advice was very high that is 4.19%. Leaving against medical advice remains a prevalent problem of health care systems, with burdens on patients' outcomes, economy, and hospitals' resources.^15^ This action leaves the patient with inadequately treated medical problems and increased risk for readmissions and mortality as shown in a study done by Choi et al.^16^ To address this problem, a patient-centered approach is crucial. This involves harmonizing patient-provider relationships and policies, as well as improving communication and counseling. Ensuring that patients feel assured about the health services provided by the hospital can help reduce the incidence of LAMA. Effective communication and counseling can help patients understand the importance of completing their treatment plans and the potential risks of leaving early.

The leading cause of death was diseases of the respiratory system accounting for 21.45% followed by diseases of liver particularly chronic disease of liver and its complications 19.54% and infective diseases 12.43% respectively. The mortality rate among the admitted patients in different wards varied from lowest in endocrine 0.82% and highest in general medicine 53.14%. Highest number of deaths was between the age 70 and 80 years. The median age of mortality among male 57 years (IQR: 44-70 years). The median age of mortality of female was 60 (IQR: 45-73 years). The highest mortality rate is seen after the age of 90 years.The fact that respiratory diseases are the leading cause of death, followed closely by liver diseases and infectious diseases, points to critical areas needing attention. Respiratory diseases' prominence suggests a need for better respiratory care and preventive measures, while the high mortality from liver diseases indicates the necessity for improved liver disease management and early intervention strategies.

The median hospital stay before death was 5 (IQR:2-9) days. The average length of hospital stays is widely recognized as a key efficiency indicator, carrying significance in clinical, financial, and operational contexts. In a study done in Nigeria, the mean duration of hospital stay for those who died (4.95 days) was significantly shorter than that for those who were discharged (12.5 days).^17^ The studies done in Nigeria and Ethiopia show similar duration of hospital stay of patients before dying.^18^ The mean duration of stay for those who died was 8 days.^19^ This metric varies across nations, exemplified by a reported range of 4.5 days in the United States to 7.2 days in Canada, contingent upon the nature of diseases treated and available healthcare services. In the context of this study, the observed average hospital stay was 6.8 days, surpassing the U.S. benchmark of 4.5 days.^20^ Furthermore, it is noteworthy that the age-adjusted average length of stay(ALOS) in Canadian hospitals during the period 2021-2022 was reported at 7.2 days.^21^ These findings underscore the necessity of considering contextual factors when assessing and comparing average hospital stays. Regional disparities and demographic factors play significant roles in influencing healthcare outcomes.

Our study has certain limitations. First, the fact that it was conducted at a single center limits the generalizability of the findings. While the results provide valuable insights into the patient population at this center, they may not be representative of broader populations with varying demographics, healthcare systems, or clinical practices. Larger, multicenter studies would be required to ensure the results apply to diverse settings and patient populations. By excluding patients admitted to the emergency department, the study may overlook critical cases and acute conditions that significantly impact overall mortality and morbidity rates. Variations in patient conditions, treatment protocols, and other factors could introduce biases that are not accounted for in this study.

## CONCLUSIONS

The higher mortality rate observed in our study compared to those in developed countries and similar rate observed in similar settings emphasizes the critical role of advanced health services in reducing mortality. Respiratory disease was the leading cause of death in hospitals which is similar to other studies done in similar settings. These findings suggest that enhancing healthcare facilities and early intervention strategies could improve patient outcomes, particularly for conditions like liver disease, which demonstrated the highest mortality rate in our study.
